# Amino Alcohol Oxidation with Gold Catalysts: The Effect of Amino Groups

**DOI:** 10.3390/ma6072777

**Published:** 2013-07-12

**Authors:** Alberto Villa, Sebastiano Campisi, Marco Schiavoni, Laura Prati

**Affiliations:** Department of Chemistry, Università degli Studi di Milano, via Golgi 19, Milano 20133, Italy; E-Mails: alberto.villa@unimi.it (A.V.); sebastiano.campisi@unimi.it (S.C.); marco.schiavoni@unimi.it (M.S.)

**Keywords:** gold catalysts, serine, ethanolamine, aminoalcohol oxidation

## Abstract

Gold catalysts have been prepared by sol immobilization using Tetrakis(hydroxymethyl) phosphonium chloride (THPC) as a protective and reducing agent or by deposition on different supports (Al_2_O_3_, TiO_2_, MgAl_2_O_4_, and MgO). The catalytic systems have been tested in the liquid phase oxidation of aminoalcohols (serinol and ethanolamine) and the corresponding polyols (glycerol and ethylene glycol). This comparison allowed us to state that the presence of amino groups has a crucial effect on the catalytic performance, in particular decreasing the durability to the catalysts, but did not substantially vary the selectivity. A support effect has been as well established.

## 1. Introduction

Amino acids are abundantly present in nature and they are obtained from hydrolized protein or, as an alternative, they are produced through fermentation processes [1]. When a single amino acid is not present in the proteins, or it cannot be obtained by biotechnology, it can be prepared by classical methods such as Gabriel synthesis [2], the Sorensen method [3], and Strecker synthesis [4]. In particular, Glycine is normally obtained from chloroacetic acid by amination with an excess of ammonia [5]. Serine is obtained by microbial/enzymatic conversion of glycine using immobilized resting cells, or crude cell extracts, by serine hydroxymethyltransferase [6]. The production of amino acids by direct oxidation of amino alcohols, using O_2_ as oxidant, in the presence of heterogeneous catalysts, represents a suitable alternative. The main problem lies on the high affinity of nitrogen for metal such as Pt or Pd, which lead to active sites blocking [7]. Gold catalyst appeared more resistant, and therefore applicable [8]. It has been shown, for example, that alaninol can be directly oxidized to alanine using Au/Al_2_O_3_ and using water as solvent under basic conditions [8]. Moreover, the authors showed that in order to obtain an active system, the addition of a base is required despite the presence of a basic amino group. More recently, the importance of the support and of the reaction conditions in the Au catalyzed oxidation of ethanolamine (EA), 2-methylaminoethanol (MEA), and 2-dimethylaminoethanol (DEA) [9] has been highlighted. The N-oxidation instead of –OH oxidation, appeared mainly ruled out by reaction conditions, whereas the N-substitution deeply influenced the reaction pathway. In particular, in the primary or secondary amines, the elimination of the amino groups is favored.

This finding showed that the role of N groups on the selectivity of the process is also fundamental. Therefore with the aim to study this aspect more in details, we carried out a comparison study between the selected amino alcohol and the polyol counterpart. Ethylene glycol oxidation was thus compared to ethanolamine oxidation, and glycerol to serinol.

## 2. Results and Discussion

**Figure 1:**
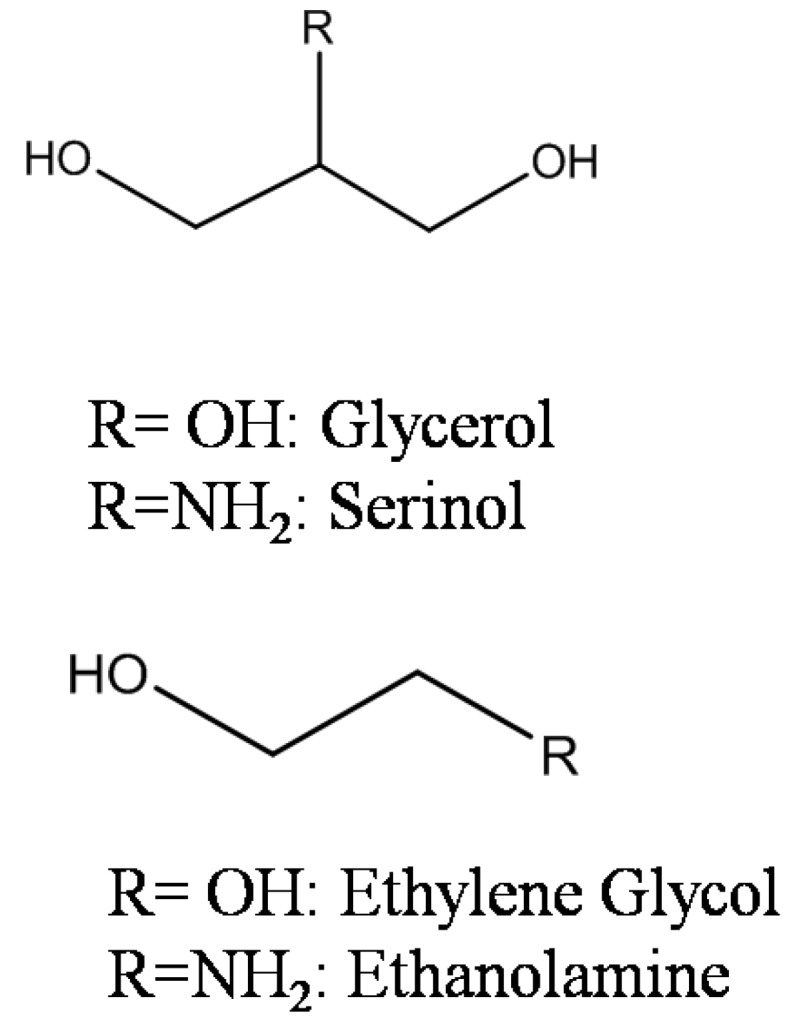
Polyols and amino alcohols investigated.

Previous report evidenced that the presence of the amino group of EA, MEA, and DEA, and corresponding oxidation products greatly affected the durability of Au based catalysts [4] depending on the substitution on nitrogen. Deactivation phenomena appeared minimized using Au catalysts prepared by sol immobilization, compared to deposition precipitation prepared ones [9]. Therefore we now investigated the effect of NH_2_ group on amino alcohol oxidation depending on the skeleton structure of the molecule. For better highlighting of the NH_2_ group effect, we compared structurally similar polyols with the corresponding amino alcohols. In this paper, the Au catalyzed oxidation of glycerol and ethylene glycol and the corresponding amino alcohols (serinol and ethanolamine) (Figure 1) has been studied. The effect of the preparation method, and of the support on the activity, selectivity, and durability, of the catalysts has also been investigated.

Au catalysts were synthesized by a previously described procedure of sol immobilization using Au-THPC protected nanoparticles (AuNPs) or, in alternative, by deposition [10,11] using different metal oxides (TiO_2_, Al_2_O_3_, MgO, MgAl_2_O_4_) as the support. TEM studies showed almost the same Au mean size for all catalyst (3–4 nm), except for Au_DP_/MgO where aggregation of Au particles has been observed resulting in mean Au size of 6.2 (Table 1). Table 1 also reported standard deviation derived from counting more than 300 particles. It should be noted that in the case of Au prepared by sol immobilization, the same Au loading, 1% wt, has been obtained. Conversely, in the case of the catalysts prepared by deposition precipitation, the loading varied accordingly to the support, as this methodology does not allow narrowly controlling this parameter (Table 1).

**Table 1:** Statistical median and standard deviation of particle size analysis for Au catalysts.

Catalyst	Statistical median (nm)	Standard deviations
1% Au_THPC_/MgAl_2_O_4_	3.8	2.3
1% Au_THPC_/MgO	2.8	1.1
1% Au_THPC_/TiO_2_	3.2	1.4
1% Au_THPC_/Al_2_O_3_	3.9	1.2
1.50% Au_DP_/MgAl_2_O_4_	2.4	0.6
0.87% Au_DP_/MgO	6.2	1.8
0.79% Au_DP_/TiO_2_	3.1	1.3
0.82% Au_DP_/Al_2_O_3_	3.9	1.2

The catalyst were tested in the selective oxidation of polyols and amino alcohols using water as solvent, alcohol/metal ratio 1000 mol/mol, pO_2_ = 3 atm, T = 50 °C, in presence of a base (4eq NaOH). These reaction conditions have been reported to maximize the selectivity to the acids and amino acids respectively [9,12].

**Figure 2:**
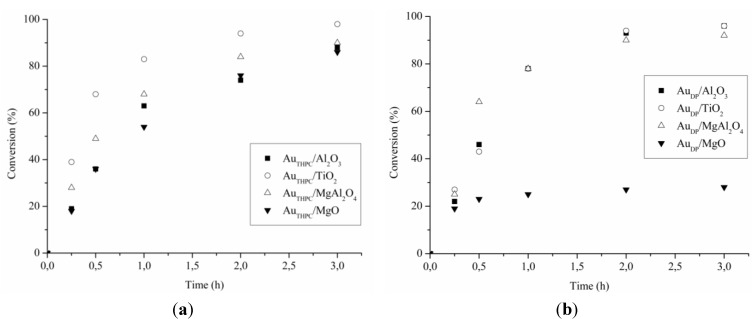
Reaction profiles for glycerol oxidation using (**a**) Au_THPC_; and (**b**) Au_DP_ catalysts.

**Figure 3:**
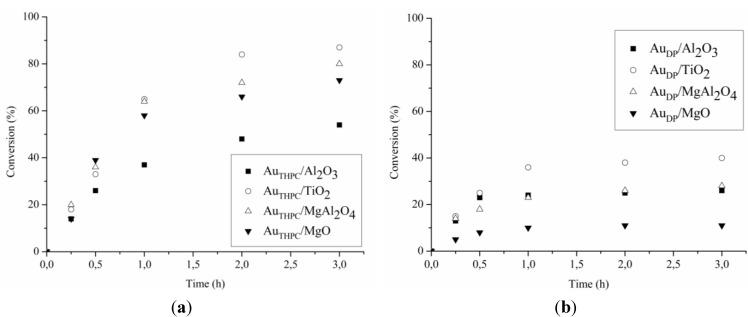
Reaction profiles for serinol oxidation using (**a**) Au_THPC_; and (**b**) Au_DP_ catalysts.

Table 2 reported the catalytic performance of Au based catalysts for glycerol and serinol oxidation (TOF). All catalyst showed better activity in the glycerol oxidation compared to serinol oxidation. Thus, the introduction of an NH_2_ group into the molecule does not have a positive effect on the catalytic performance. The analysis of the reaction profiles (Figure 2 and Figure 3) suggested that deactivation never occurs in glycerol oxidation, except for Au_DP_/MgO, whereas it is present in serinol oxidation with almost all catalysts. In fact, by recycling the catalyst at the end of the reaction we did not observe any loss of activity in the case of glycerol, whereas in the case of serinol, the same activity of fresh catalysts has not been revealed. Deactivation occurring in serinol oxidation is more evident in the case of Au_DP_ catalysts, which are never able to reach a serinol conversion higher than 40% (Figure 3b). These data confirmed that the partial poisoning effect of the NH_2_ group on the catalyst became more important in the case of DP prepared samples.

**Table 2:** Comparison of Au catalyst activities in glycerol and serinol oxidation.

Catalyst ^[a]^	Glycerol				Serinol	
	TOF (h^−1^) ^[b]^	Selectivity (%) ^[c]^			TOF (h^−1^) ^[b]^	Selectivity to Serine ^[c]^
		Glycerate	Glycolate	Tartronate		
1% Au_THPC_/MgAl_2_O_4_	1089	58	24	13	600	41
1% Au_THPC_/MgO	686	60	25	4	440	46
1% Au_THPC_/TiO_2_	1447	63	27	8	590	33
1% Au_THPC_/Al_2_O_3_	495	62	27	1	431	30
1.50% Au_DP_/MgAl_2_O_4_	624	57	24	16	435	43 ^[^^d^^]^
0.87% Au_DP_/MgO	376	74 ^[^^d^^]^	18 ^[^^d^^]^	2 ^[^^d^^]^	68	40 ^[^^d^^]^
0.79% Au_DP_/TiO_2_	692	61	28	6	520	28 ^[^^d^^]^
0.82% Au_DP_/Al_2_O_3_	580	60	22	4	480	27 ^[^^d^^]^

[a] Reaction condition: alcohol/metal 1000/1 (mol/mol), 4eq NaOH, 50 °C, pO_2_ 3 atm, 1250 rpm; [b] TOF calculated after 15 min of reaction based on the total metal loading; [c] Selectivity at 50% conversion; [d] Selectivity at 20% conversion.

Therefore, the better general performance of Au_THPC_ in comparison to Au_DP_ catalysts in glycerol and serinol oxidation can be described in terms of a better resistance to deactivation of the catalyst prepared by sol immobilization. Likely, the protective layer is able to limit the adsorption of the amino group on the active site. The worst catalytic performance was showed by Au_DP_/MgO compared to all the other catalysts can be ascribed to bigger particles (mean size 6 nm instead 3–4).

The support appeared to play an essential role in determining the activity of the catalytic system as well as the resistance to deactivation. Indeed, comparing the different supports, TiO_2_ appeared the best choice regardless the preparation method. Indeed, TiO_2_ based catalysts are not only the most active in the glycerol and serinol oxidation, but they are also able to minimize the deactivation phenomena present using the other supports.

**Scheme 1:**
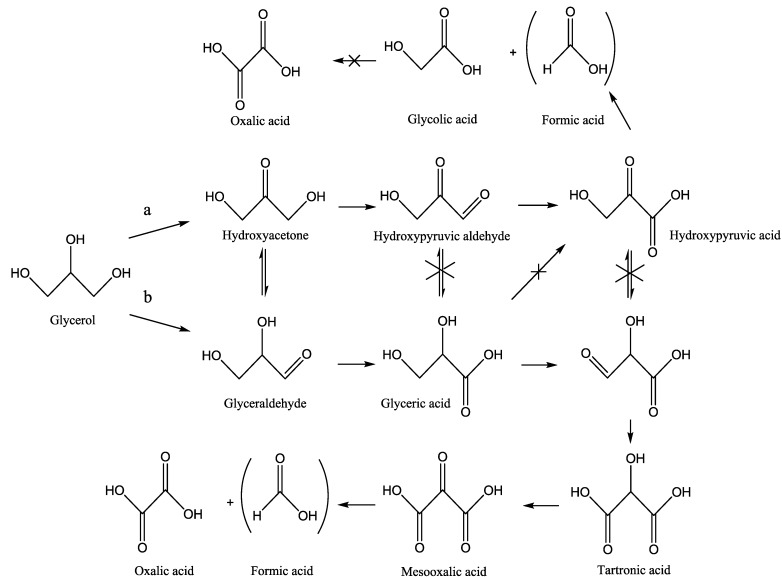
Reaction scheme for glycerol oxidation from Reference [13].

In the glycerol oxidation the catalyst preparation method, as well as the support, did not show an obvious effect on the selectivity. Indeed, in all cases the selectivity to glyceric acid is similar (58%–62%) with glycolic acid (24%–28%) as main by-product (Scheme 1). In all cases, the selectivity did not significantly change with the conversion. As expected, Au_DP_/MgO showed a higher selectivity to glyceric acid (74%) than the other catalysts according to the literature, reporting that bigger Au particles are more selective to glyceric acid [13,14,15,16,17].

**Scheme 2:**
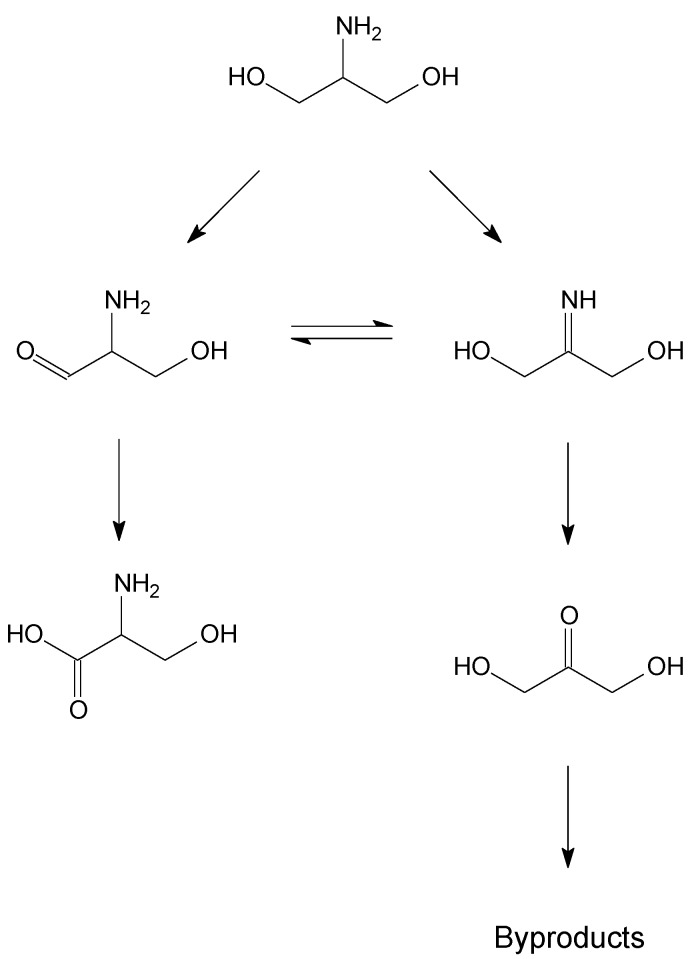
Reaction scheme for serinol oxidation.

In the oxidation of serinol, it can be observed that all catalyst showed a low selectivity to serine (27%–46%) with the formation of different by-products, as shown in Scheme 2. Differently to glycerol, the support seems to play an important role. Indeed, independently to the preparation method used, Au supported on more basic supports (MgO, MgAl_2_O_4_) showed better selectivity (40%–46%) than when supported on Al_2_O_3_ and TiO_2_ (27%–33%).

**Figure 4:**
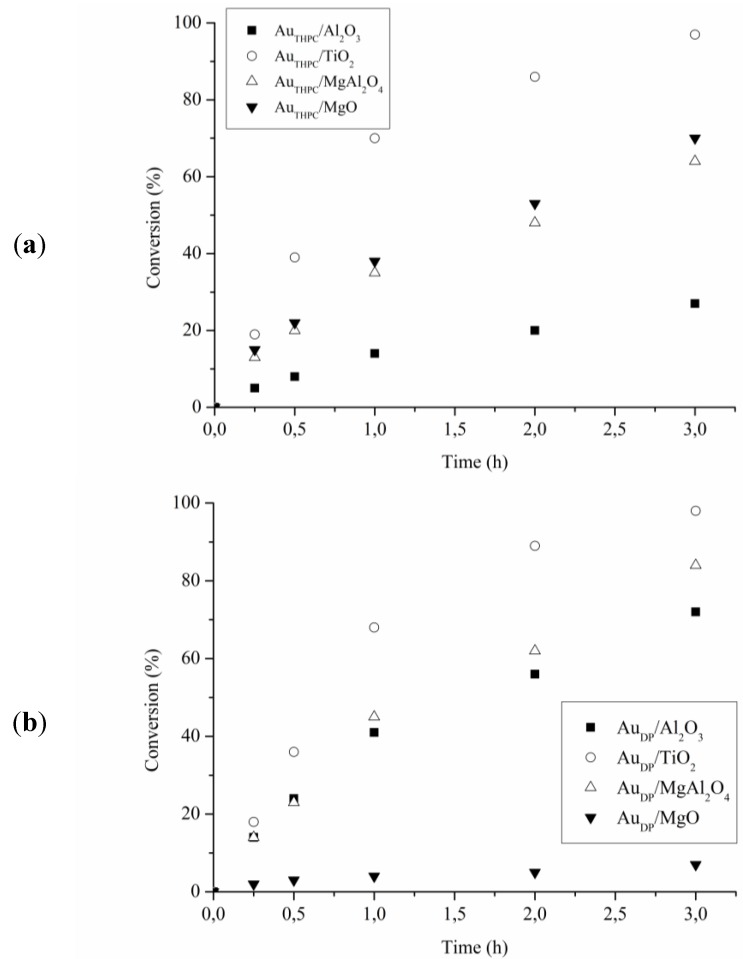
Reaction profiles for ethylene glycol oxidation using (**a**) Au_THPC_; and (**b**) Au_DP_ catalysts.

**Figure 5:**
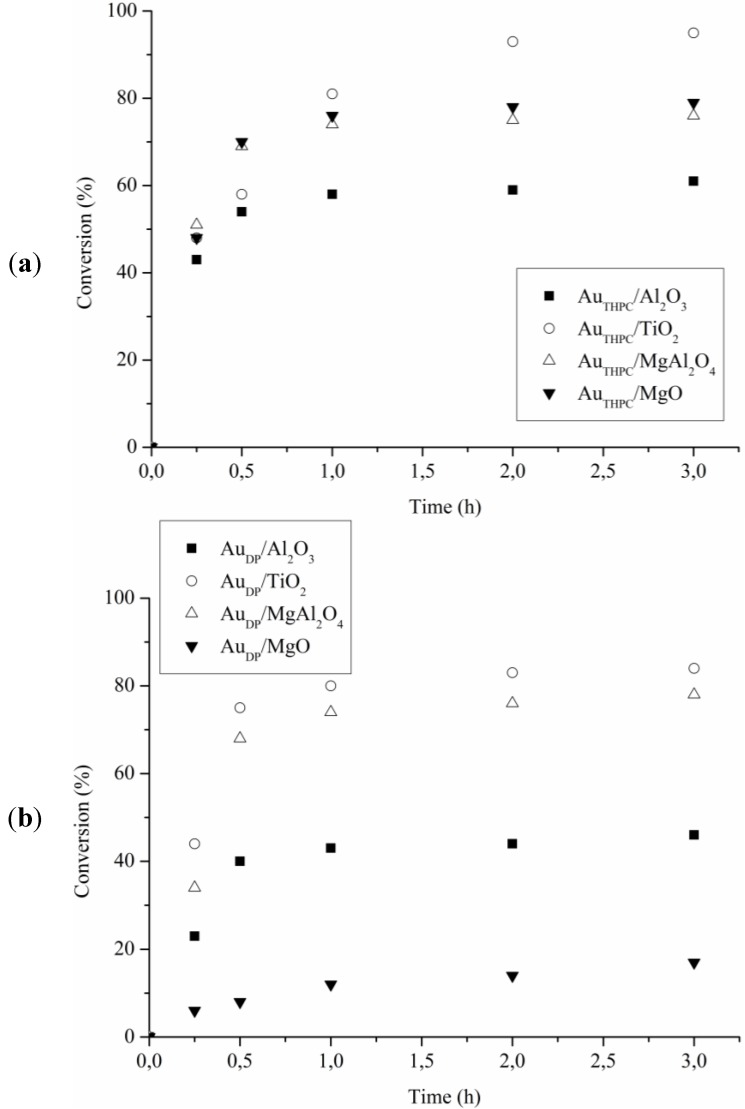
Reaction profiles for ethanolamine oxidation using (**a**) Au_THPC_; and (**b**) Au_DP _catalysts.

To further evaluate the activity of the catalytic materials the oxidation of ethylene glycol (Figure 4) and ethanolamine (Figure 5) was undertaken. Surprisingly in this case all catalyst showed a better performance in the oxidation of ethanolamine than in the oxidation of the corresponding ethylene glycol (Table 3). Indeed, all catalysts, except Au_DP_/Al_2_O_3_ and Au_DP_/MgO, showed a conversion between 35% and 50% after 15 min (Figure 5) in the ethanolamine oxidation, whereas the same ones showed an activity always lower than 20% in the ethylene glycol oxidation (Figure 4). However, as in the previous case, strong deactivation phenomena have been observed after 30 min of reaction in the amino alcohol oxidation (Figure 5) when catalyst reached maximum conversion. Conversely, a more linear activity in the oxidation of ethylene glycol (Figure 4) was observed. In these latter cases, in fact, it was possible to reach full conversion just by prolonging the reaction time. In particular, Au/TiO_2_ showed constant activity allowing completing ethylene glycol conversion in less than three hours. In addition, in these cases, the absence of deactivation phenomena was revealed by recycling the catalyst.

**Table 3:** Comparison of Au catalyst activities in ethylene glycol and Ethanolamine oxidation.

Catalyst ^[a]^	Ethylene glycol			Ethanolamine	
	TOF (h^−1^) ^[b]^	Selectivity (%) ^[c]^		TOF (h^−1^) ^[b]^	Selectivity to Glycine ^[c]^
		Glycolate	Oxalate		
1% Au_THPC_/MgAl_2_O_4_	576	98	2	2991	88
1% Au_THPC_/MgO	461	97	3	2604	53
1% Au_THPC_/TiO_2_	711	97	3	2693	78
1% Au_THPC_/Al_2_O_3_	79	n.d.	n.d	2124	96
1.50% Au_DP_/MgAl_2_O_4_	553	95	5	1698	92
0.87% Au_DP_/MgO	39	n.d.	n.d	273	n.d
0.79% Au_DP_/TiO_2_	590	95	5	2293	91
0.82% Au_DP_/Al_2_O_3_	448	95	4	908	87

[a] Reaction condition: alcohol/metal 1000/1 (mol/mol), 4eq NaOH 50 °C, pO_2_ 3 atm, 1250 rpm; [b] TOF calculated after 15 min of reaction based on the total metal loading; [c] Selectivity at 50% conversion.

On the basis of these data we could conclude that the amino group in the ethanolamine has a different influence on the catalytic performance compared to serinol. Apparently, the introduction of basic amino groups is able to enhance the initial catalytic activity, affecting the durability of the catalyst. A possible explanation can lie in the different reactant structure of ethanolamine and serinol, being the NH_2_ group in this latter one more hindered, thus was less accessible to the active site.

**Scheme 3:**
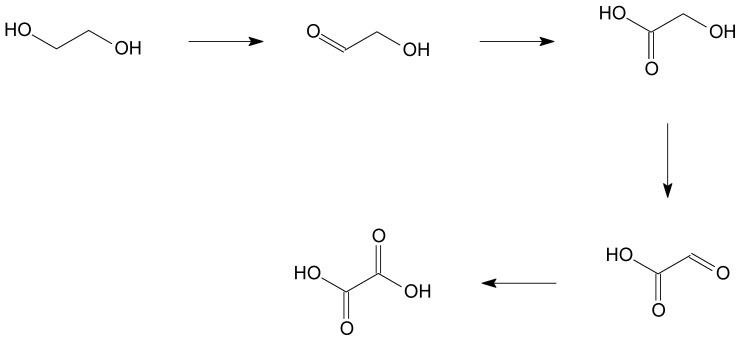
Reaction scheme for ethylene glycol oxidation.

**Scheme 4:**
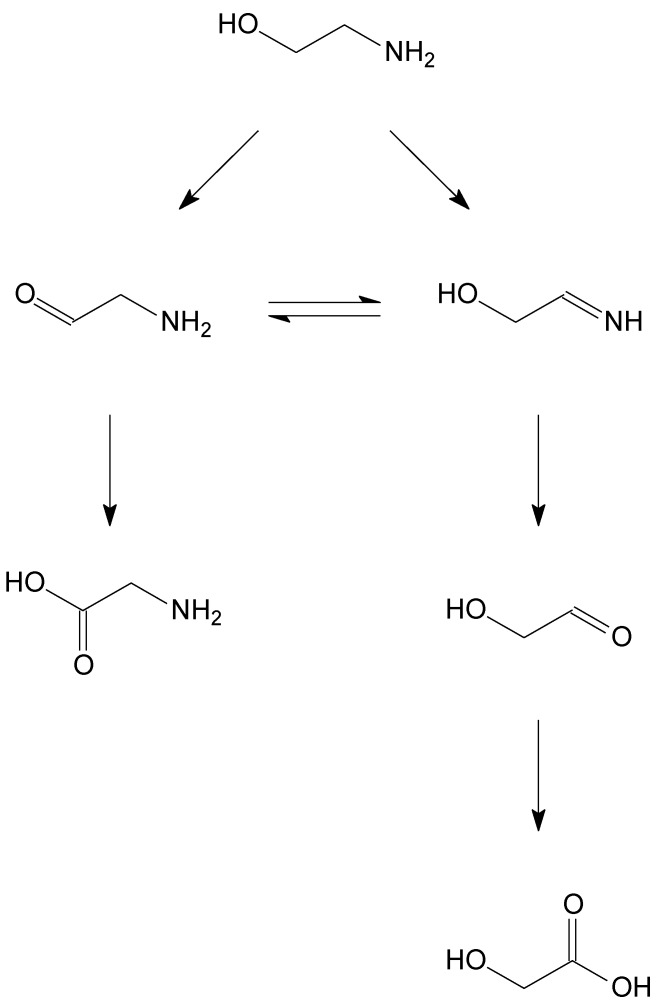
Reaction scheme for ethanolamine oxidation from Reference [9].

From a selectivity point of view, all catalyst showed high selectivity to glycolic acid (>95%) with formation of only a small amount of oxalic acid, deriving from the subsequent oxidation of the second alcohol group (Scheme 3). Conversely, in ethanolamine oxidation, deamination process lowered the selectivity to glycine, ranging from 53% to 95%, glycolic acid becoming the main by-product (Scheme 4).

## 3. Experimental Section

Gold of 99.99% purity in sponge from Fluka was used. Gamma-Al_2_O_3_ from Condea (SA = 90 m^2^/g), MgO from Merck (SA = 35 m^2^/g), TiO_2_ from Degussa (SA = 50 m^2^/g), and MgAl_2_O_4_ spinel was reported in Reference [18] (SA = 150 m^2^/g). A reacting solution was prepared by dissolving aluminum nitrate (0.1 M), magnesium nitrate (0.08 M), and urea (1.8 M) in distilled water. The pH of the starting solution was adjusted to 2 with nitric acid and then the solution was heated at 90 °C under magnetic stirring for 24 h. The precipitate was washed three times with distilled water using magnetic stirring and centrifugation, and dried at 100 °C for 16 h. The so-synthesized powder was calcined in air for 1 h at 900 °C.

Tetrakis(hydroxymethyl)phosphonium chloride (THPC, 80% solution) from Aldrich was used. NaOH of the highest purity available and urea (purity > 99%) were from Fluka. Gaseous Oxygen from SIAD was 99.99% pure. Ethylene glycol (>99.5% pure), ethanolamine (purity > 99.0%), glycerol (86%–88% solution), 2-amino-1,3-propanediol (serinol, 99% purity), and 3-amino-1,2-propanediol (>98.0% pure) from Fluka were used in oxidation experiments. DSS (3-trimethylsilyl-1-propanesulfonic acid, sodium salt), o-phthaldialdehyde (OPA), 2-mercaptoethanol, and all the products used as standard samples were from Fluka.

Au_THPC_. Sols generated in the presence of the THPC/NaOH system were prepared as reported by Grunwaldt* et al.* [10]. A freshly prepared 0.05 M solution of THPC was added to a 10^−3^ M solution of NaOH. After a few minutes, HAuCl_4_ 10^−3^ M was added dropwise, yielding a brown metallic sol. Within a few minutes of sol generation, complete reduction of Au(III) was checked by UV analysis. Then, the sol was immobilized by adding the support under vigorous stirring. The amount of support was calculated for having a final gold loading of 1% wt. After 2 h the slurry was filtered and the catalyst washed thoroughly with distilled water; it was then used in the wet form.

Au_DP_. The catalysts were prepared by the deposition-precipitation method reported by Louis* et al.* [11] using urea as the precipitating agent. The support (1 g) was added to 100 mL of an aqueous solution of HAuCl_4_ (100 ppm of Au) and urea (0.42 M). The suspension, thermostated at 80 °C, was vigorously stirred for 4 h, until pH 7.20 was reached. The slurry was then filtered, washed thoroughly with water, dried at 80 °C for 2 h, and then calcined in air at 450 °C for 4 h or reduced under H_2_ at 180 °C for 2 h.

Morphology and microstructures of the catalysts were characterized in a Philips CM200 FEG electron microscope, operating at 200 kV and equipped with a Gatan imaging filter, GIF Tridiem.

Powder samples of the catalysts were ultrasonicated in ethanol and dispersed on copper grids covered with a holey carbon film. The particle size distribution for each catalyst was determined by measuring the mean diameter of over 300 particles from different areas. Each size distribution can be fitted by a log-normal function.

Reactions were carried out in a thermostated glass reactor (30 mL), provided with an electronically controlled magnetic stirrer, connected to a large reservoir (5000 mL) containing oxygen at 300 kPa. The oxygen uptake was followed by a mass-flow controller connected to a PC through an A/D board, plotting a flow/time diagram. Substrate (0.3 M solution), NaOH (substrate/NaOH 1:4 eq), and gold catalyst (substrate/metal = 1000 mol/mol) were mixed in distilled water (total volume 10 mL). The reactor was pressurized at 300 kPa with O_2_ and thermostated at 50 °C. After an equilibration time of 10 min, the reaction was started by stirring. Samples were periodically taken and analyzed by HPLC or ^1^H-NMR spectroscopy. Analyses of polyols and oxidation products were performed on a Varian 9010 HPLC equipped with a Varian 9050 UV (210 nm) and a Waters R.I. detector in series. A Varian MetaCarb H Plus column (300 mm × 7.8 mm) was used with aqueous H_3_PO_4_ 0.1% wt/wt (0.4 mL/min) as the eluent. Products were recognized by comparison with authentic samples. Ethanolamine and oxidation products were analyzed as described in [9]. Analyses were performed on a Bruker AC 300 NMR spectroscope. 100 μL of DSS (internal standard) solution in D_2_O (20 mg/mL) were added to each sample (500 μL). Proton NMR spectra of the samples were recorded with low power PRESAT water suppression, in order to minimize signal distortions. Products were recognized by comparison with authentic samples, and their concentrations were determined by comparing products signals areas to standard signal area.

Serinol and oxidation products were analyzed on a Varian 9010 HPLC equipped with a Varian 9050 UV (230 nm). A ChromSpher 5 C18 column (200 mm × 3.0 mm) was used with (A) 0.025 M phosphate buffer pH 7.9 + 0.75% THF, (B) methanol:water (60:40) as the eluents. Samples of the reaction mixture were diluted with distilled water and derivatized with o-phthaldialdehyde as previously reported [9]. Products were recognized by comparison with authentic samples.

## 4. Conclusions

Au based catalysts have been tested in aminoalcohol oxidation to evaluate the impact of amino group on activity and selectivity. With this aim, differently prepared catalysts were used in glycerol/serinol and ethylene glycol/ethanolamine oxidation. Catalysts prepared by the sol-immobilisation technique appear more active and resistant than the ones prepared by deposition-precipitation. The substitution of –OH group with –NH_2_ in the molecule seems to have an opposite trend on the initial activity (measured as TOF) moving from glycerol/serinol to ethyleneglycol/ethanolamine. Indeed all catalyst was more active in glycerol than serinol and *vice versa* for ethyleneglycol/ethanolamine. The structure of the molecule, and in particular the position of the NH_2_ group and its possibility to adsorb on the active site, is a key point. Indeed the less sterical hindrance of the –NH_2_ group of ethanolamine easier coordinate to the catalyst active site than serinol, resulting in a stronger decrease of the durability of the catalysts.

The support seems to have also a strong influence on the catalytic activity being TiO_2_ based catalysts more active in all the cases than Au on other oxides. This strong support effect could be addressed to both geometric and electronic effect. Indeed, it was reported that TiO_2_ could stabilize partial oxidative Au species that could imply a different mechanism with respect to Au(0).

From selectivity point of view serinol and ethanolamine behave differently. In the case of serinol, quite low selectivity to amino acid was obtained (<46%) whereas reasonably good results can be obtained in the case of ethanolamine (<96%). The selectivity typically depends on the support and appeared quite independent from the catalyst preparation method.
